# *Epilobium angustifolium* L. Extracts as Valuable Ingredients in Cosmetic and Dermatological Products

**DOI:** 10.3390/molecules26113456

**Published:** 2021-06-07

**Authors:** Anna Nowak, Martyna Zagórska-Dziok, Paula Ossowicz-Rupniewska, Edyta Makuch, Wiktoria Duchnik, Łukasz Kucharski, Urszula Adamiak-Giera, Piotr Prowans, Norbert Czapla, Piotr Bargiel, Jan Petriczko, Marta Markowska, Adam Klimowicz

**Affiliations:** 1 Department of Cosmetic and Pharmaceutical Chemistry, Pomeranian Medical University in Szczecin, PL-70111 Szczecin, Poland; wiktoria.duchnik@pum.edu.pl (W.D.); lukasz.kucharski@pum.edu.pl (Ł.K.); adam.klimowicz@pum.edu.pl (A.K.); 2 Department of Technology of Cosmetic and Pharmaceutical Products, Medical College, University of Information Technology and Management in Rzeszow, Sucharskiego 2, PL-35225 Rzeszów, Poland; mzagorska@wsiz.rzeszow.pl; 3 Department of Chemical Organic Technology and Polymeric Materials, Faculty of Chemical Technology and Engineering, West Pomeranian University of Technology in Szczecin, PL-70322 Szczecin, Poland; possowicz@zut.edu.pl (P.O.-R.); emakuch@zut.edu.pl (E.M.); 4 Department of Pharmacokinetics and Therapeutic Drug Monitoring, Pomeranian Medical University in Szczecin, PL-70111 Szczecin, Poland; uadamiak-giera@pum.edu.pl; 5 Department of Plastic, Endocrine and General Surgery, Pomeranian Medical University in Szczecin, PL-72010 Police, Poland; piotr.prowans@pum.edu.pl (P.P.); norbert.czapla@pum.edu.pl (N.C.); piotr.bargiel@pum.edu.pl (P.B.); jan.petriczko@pum.edu.pl (J.P.); markowskamh@gmail.com (M.M.)

**Keywords:** fireweed, antioxidants, anti-inflammatory properties, antiaging properties, collagenase, penetration skin, phenolic acids, hydrogel, emulsion

## Abstract

*Epilobium angustifolium* L. is a popular and well-known medicinal plant. In this study, an attempt to evaluate the possibility of using this plant in preparations for the care and treatment of skin diseases was made. The antioxidant, antiaging and anti-inflammatory properties of ethanolic extracts from *Epilobium angustifolium* (FEE) were assessed. Qualitative and quantitative evaluation of extracts chemically composition was performed by gas chromatography with mass spectrometry (GC-MS) and high-performance liquid chromatography (HPLC). The total polyphenol content (TPC) of biologically active compounds, such as the total content of polyphenols (TPC), flavonoids (TFC), and assimilation pigments, as well as selected phenolic acids, was assessed. FEE was evaluated for their anti-inflammatory and antiaging properties, achieving 68% inhibition of lipoxygenase activity, 60% of collagenase and 49% of elastase. FEE also showed high antioxidant activity, reaching to 87% of free radical scavenging using 2,2-diphenyl-1-picrylhydrazyl (DPPH) and 59% using 2,2′-azinobis(3-ethylbenzothiazoline-6-sulfonic acid) (ABTS). Additionally, in vitro penetration studies were performed using two vehicles, i.e., a hydrogel and an emulsion containing FEE. These studies showed that the active ingredients contained in FEE penetrate through human skin and accumulate in it. The obtained results indicate that *E. angustifolium* may be an interesting plant material to be applied as a component of cosmetic and dermatological preparations with antiaging and anti-inflammatory properties.

## 1. Introduction

Nowadays, there is growing interest in the search for novel, effective, and safe dermatological preparations containing active ingredients with multiple effects. One of the potential sources of biologically active substances used in the treatment of various skin diseases are plants. Due to the abundance of secondary metabolites they contain, plant extracts can play multiple roles, such as antioxidant, anti-inflammatory, and antiaging [1]. Furthermore, dermatological and cosmetic products containing mainly “natural” ingredients are perceived as safer compared to “synthetic” ingredients [1,2]. It is well-known that plants can produce natural antioxidant compounds that could control oxidative stress [3]. Reactive oxygen species (ROS) act primarily by several important molecular pathways that play essential roles in diverse pathologic processes, including inflammatory responses. The skin possesses an array of defense mechanisms that interact with toxicants to prevent their harmful effect. Unfortunately, although highly effective, these defenses have limited capacity and can foster dermatological diseases. Free radicals play a crucial role as they impact the condition of the skin. They can induce and maintain cutaneous inflammation or affect the development of infection [4]. For example, common bacterial infections in the skin and underlying tissues are dependent on oxidative stress. More ROS are released in inflammation, which protects the body against microbes [5]. Fireweed (*Epilobium angustifolium* (L.) Holub) (Onagraceae) is a well-known medicinal plant that occurs naturally on many continents [6,7]. This plant has long been used to treat inflammation and gastrointestinal disorders and to improve wound healing, swelling, and skin sores [8,9]. In recent years, there has been a growing interest in this plant in treating skin diseases, where it is used as an ingredient in cosmetic and dermatological preparations. It is mainly due to its antibacterial, anti-inflammatory, and antioxidant properties [3,10,11]. For example, Karakaya reports intense wound healing due to the antioxidant effect of *E. angustifolium* [9]. The extract of this plant is effective in the treatment of as eczema, acne, minor burns, skin rashes, and ulcers. North American Indians have used *Epilobium* species to treat infected wounds [12]. On the other hand, Zagórska-Dziok mentioned *E. angustifolium* as a potential cosmetic plant with antiaging properties confirmed by the cytoprotective effect on skin cells, keratinocytes as well as fibroblasts [3]. *E. angustifolium* undoubtedly owes its pharmacological effect to its rich composition, including the content of valuable polyphenols, including phenolic acids such as chlorogenic acid (ChA), gallic acid (GA), 4-hydroxybenzoic acid (4-HB), 3,4-dihydroxybenzoic acid (3,4-DHB), or caffeic acid (CA) [10]. These compounds show primarily antioxidant activity [13] in preparations used on mucous membranes and skin [14]. Estimating the skin penetration of plant substances is an integral part of the research due to some limited penetration through the stratum corneum (SC), the important barrier for the permeation of the exogenous compounds to the skin [15]. Therefore, it is also essential to select the appropriate vehicle from which the active ingredients contained in the plant extract will be released. In the previous work, we found that some phenolic acids in the ethanolic extract of *E. angustifolium* penetrated human skin and accumulate in it [10]. 

In this study, we decided to examine the antioxidant, antiaging, and anti-inflammatory activity of ethanol extracts from *E. angustifolium* (FEE), as well as the penetration of selected phenolic acids from hydrogel and emulsion, often used in dermatology or cosmetology.

## 2. Results

### 2.1. Determination of Biologically Active Compounds

**Figure 1:**
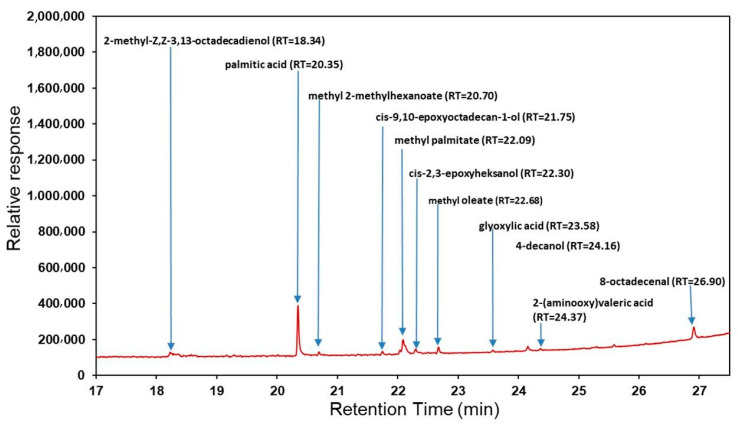
GC-MS chromatogram of the ethanolic extracts of *E. angustifolium* (FEE).

Figure 1 presents the GC-MS chromatogram of FEE. The following compounds were identified: 2-methyl-Z,Z-3,13-octadecadienol, palmitic acid, methyl 2-methylhexanoate, cis-9,10-epoxyoctadecan-1-ol, methyl palmitate, cis-2,3-epoxyheksanol, methyl oleate, glyoxylic acid, 4-decanol, 2-(aminooxy)valeric acid, and 8-octadecenal. 

**Figure 2:**
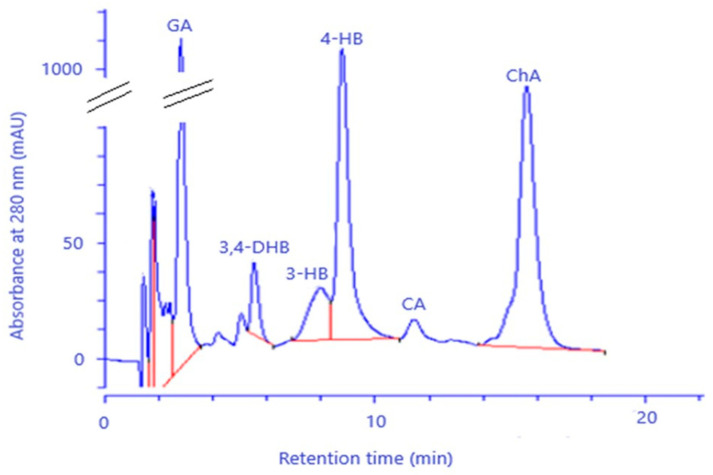
Chromatogram of phenolic acid identified in the ethanol extracts from *E. angustifolium* (FEE): GA—gallic acid; 3,4-DHB—3,4-dihydroxybenzoic acid, 4-HB—4-hydroxybenzoic acid, 3-HB—3-hydroxybenzoic acid, ChA—chlorogenic acid, and CA—caffeic acid.

The HPLC method was used to identify and quantify selected phenolic acids in FEE (Figure 2), whereas the quantitative composition of phenolic acids in the extracts determined by HPLC is presented in Table 1. The following phenolic acids were found: ChA, GA, 4HB, 3-HB, 3,4-DHB, and CA. The main compounds in analyzed extracts are GA (113.07 ± 5.03 mg/100 mL), 4-HA (29.72 ± 0.25 mg/100 mL), and ChA (22.03 ± 0.19 mg/100 mL) (Table 1).

FEE was characterized by very high total polyphenol and total flavonoid contents, amounting to 22.15 ± 0.13 mg GA/g DWE for the total polyphenol content and 3.37 ± 0.01 mg RR/g DWE for the total flavonoid content. In comparison, the chlorophyll a + b content was 0.74 ± 0.02 mg/g DWE (Table 2).

### 2.2. Assessment of Antioxidant Activity

#### DPPH Radical Scavenging Assay

**Figure 3:**
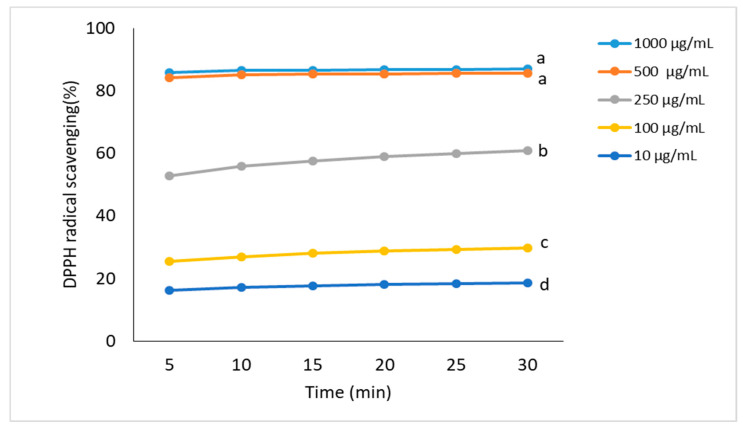
Kinetics of the antioxidant activity changes in the DPPH solutions in the presence of various concentrations of ethanolic extracts from *E. angustifolium* (FEE). Values are the mean of three replicate determinations (*n* = 3). Different letters—values are significantly different, α < 0.05.

**Figure 4:**
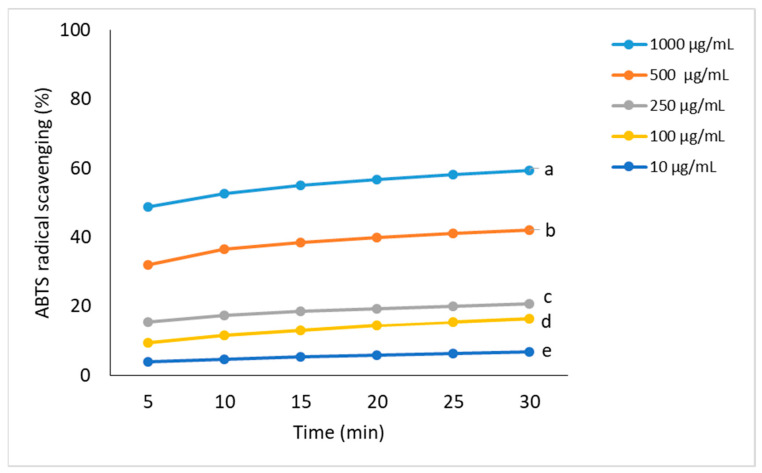
Kinetics of the antioxidant activity changes in the ABTS solutions in the presence of various concentrations of ethanolic extracts from *E. angustifolium* (FEE). Values are the mean of three replicate determinations (*n* = 3). Different letters—values are significantly different, α < 0.05.

Figure 3 and Figure 4 present the antioxidant activity of FEE, expressed as a percentage of free radical scavenging. The analyzed extracts exhibited high antioxidant properties, confirmed by the DPPH and ABTS methods. The tests were carried out in the concentration range from 10 μg/mL to 1000 μg/mL for 30 min, whereas the measurements were performed at five-minute intervals. For most of the tested concentrations, a slight increase in antioxidant activity was observed over time. In the DPPH method, a similar antioxidant activity was observed for 1000 μg/mL and 500 μg/mL, ranging from 84% to 86%. No statistically significant differences were found between these concentrations. However, due to the high antioxidant potential of the extract, the concentration of 500 µg/mL seemed to be sufficient. When considering the lowest used FEE concentration, its ability to reduce DPPH maintained a low 17% level. In the next-used concentration (100  μg/mL), it was about 10% higher (Figure 3). 

In the case of the ABTS method, the most increased antioxidant activity was demonstrated for the concentration of 1000 μg/mL (59%). At the lowest concentration used of FEE, its ability was increased from 3.8% to 6.8%. Like the DPPH method, there was an increase of about 10% at the next concentration (Figure 4). 

### 2.3. Assessment of Anti-Collagenase and Anti-Elastase Activity 

**Figure 5:**
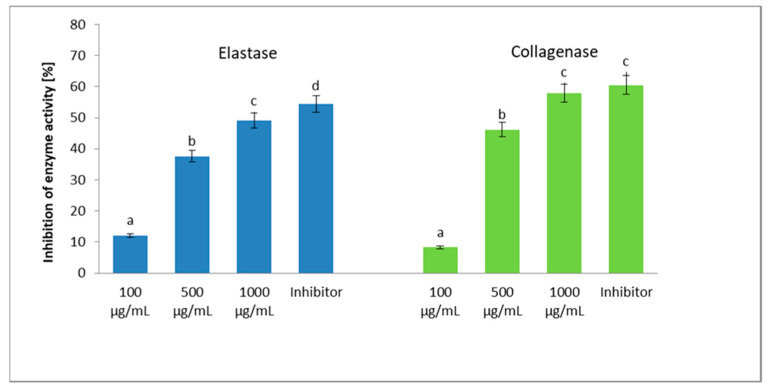
Influence of ethanolic extracts from *E. angustifolium* (FEE) on the elastase and collagenase inhibitions. SPCK for elastase and 1,10-phenanthroline for collagenase were used as the control inhibitors. Data are the mean ± SD of three independent experiments, each of which consisted of two replicates per treatment group. Vertical lines represent the standard deviation. Different letters—values are significantly different, α < 0.05.

Figure 5 presents the influence of FEE on elastase and collagenase inhibition. The extract was used in three concentrations—namely 100, 500, and 1000 µg/mL—while (succinyl-alanyl-alanyl-prolyl-valine chloromethylketone (SPCK) for elastase and 1,10-phenanthroline for collagenase were used as the control inhibitors. The highest effect was obtained using the extract at the concentration of 1000 µg/mL, in which an inhibition of the elastase activity by 49.1% and collagenase by 59.8% was achieved. In the collagenase inhibition, no significant statistical differences were found between the FEE highest concentration and the inhibitor used (Figure 5).

### 2.4. Assessment of Anti-Inflammatory Potential 

**Figure 6:**
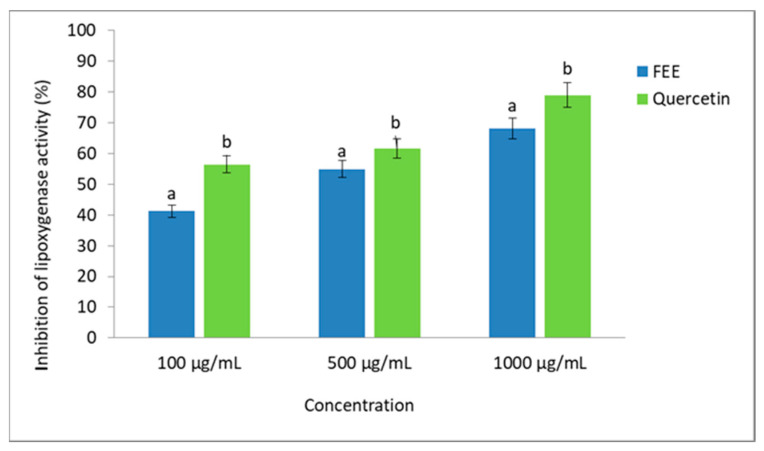
Influence of ethanolic extracts from *E. angustifolium* (FEE) on lipoxygenase inhibition. Quercetin was used as a control inhibitor. Data are the mean ± SD of three independent experiments, each of which consisted of two replicates per treatment group. Vertical lines represent the standard deviation. Different letters—values are significantly different, α < 0.05.

**Figure 7:**
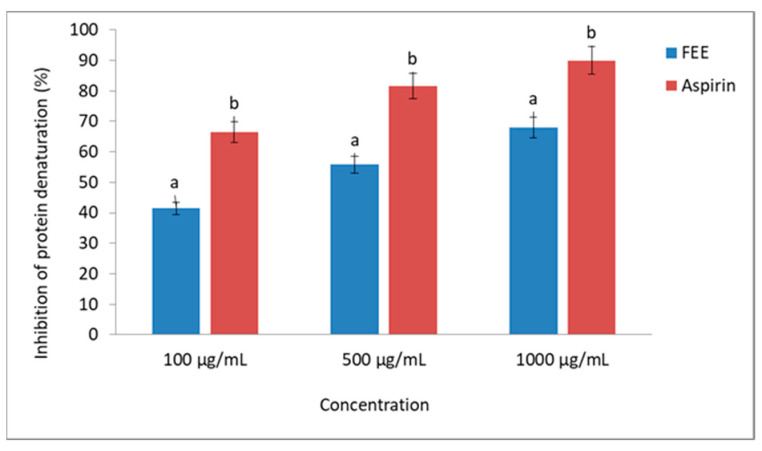
Effect of ethanolic extracts from *E. angustifolium* (FEE) on the inhibition of protein (BSA) denaturation. Acetylsalicylic acid (aspirin) was used as a control inhibitor. Data are the mean ± SD of three independent experiments, each consisting of three replicates per treatment group. Vertical lines represent the standard deviation. Different letters—values are significantly different, α < 0.05.

Figure 6 presents the influence of FEE on lipoxygenase inhibition. Quercetin was used as a control inhibitor, whereas Figure 7 illustrates the effect of FEE on bovine albumin serum (BSA) denaturation. In this case, acetylsalicylic acid (Aspirin) was used as a control inhibitor. The effect of the extract on the activity of lipoxygenase was dose-dependent. The most substantial inhibition was obtained for the extract at a concentration of 1000 µg/mL, where it reached 68.2%, while at a concentration of only 100 µg/mL, the inhibition reached over 40% (Figure 6). The conducted analyses showed that the 1000 µg/mL concentration of FEE inhibited BSA denaturation by 67.7%, while at a concentration of 100 µg/mL, the inhibition reached over 40% (Figure 7).

### 2.5. Skin Penetration

**Figure 8:**
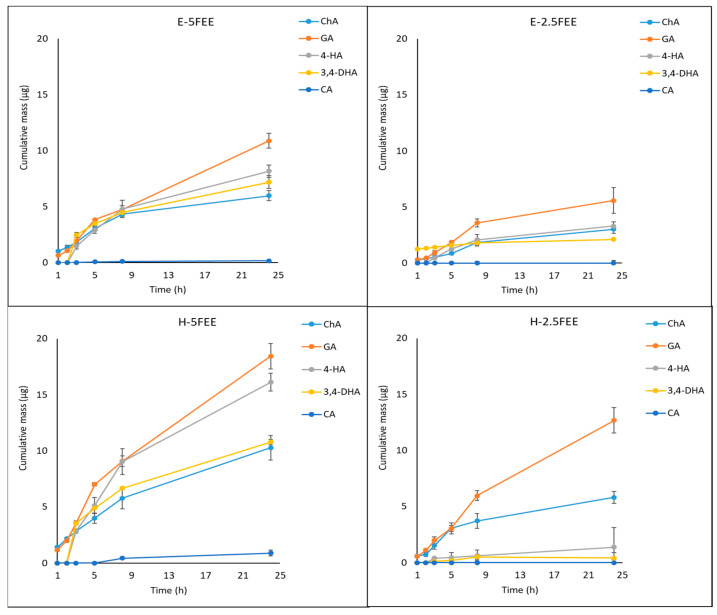
The cumulative mass of phenolic acids in the acceptor fluid during the 24-h penetration after the application of dry ethanolic extracts from *E. angustifolium* (FEE) in two vehicles. Vertical lines represent the standard deviation, *n* = 3.

Table 3 summarizes the content of selected phenolic acids in the acceptor fluid collected after 24-h penetration. The cumulative mass of phenolic acids in the acceptor fluid, considering all time points, is presented in Figure 8. The FEE (2.5% and 5%) was placed in two vehicles, namely a hydrogel and emulsion. The penetration was dependent on the vehicle used, as well as the concentration of the plant extract. In the case of H-5FEE, the cumulative mass of phenolic acids in the acceptor fluid was significantly higher than the other vehicles. Among the studied phenolic acids, GA and 4-HB penetrated to a higher degree. The cumulative amounts of these acids penetrated during the 24-h study with H-5FEE were 18.44 ± 1.13 and 16.12 ± 0.80 µg, respectively. A similar tendency was observed in the case of other vehicles. However, the cumulative mass in the acceptor fluid of GA, ChA, and 4-HB did not differ significantly between H-2.5FEE and E-5FEE (Table 3). The highest permeation parameters were observed from the hydrogel as a vehicle. The highest rate of penetration, evaluating 1.63, 1.19, and 1.09 μg·cm^−2^·h^−1^, were observed for 4-HB, GA, and ChA from H-5FEE, respectively. The lowest steady-state flux was for 3-HB. Meanwhile, CA was only detectable after an eight-hour permeation study from H-5FEE (Table 4).

**Figure 9:**
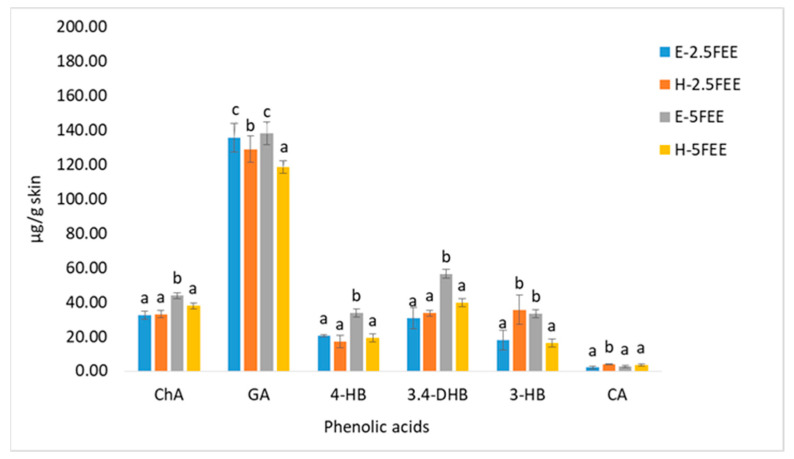
The content of phenolic acids in the solution obtained after the skin extraction collected after 24-h penetration. Vertical lines represent the standard deviation, *n* = 3. Different letters—values are significantly different, α < 0.05.

Figure 9 shows the accumulation of phenolic acids in the skin. The determination of phenolic acids was performed in the fluid obtained after skin extraction, collected after the end of the 24-h penetration. All analyzed phenolic acids accumulated in the skin. GA was accumulated in the highest amount, from 118.38 µg/g skin after the H-5FEE application to 138.01 µg/skin after the E-5FEE application. Significant differences were observed depending on the vehicle used. After applying E-5FEE on it, the skin accumulated significantly more ChA, 4-HB, and 3,4-DHB compared to the other vehicles (Figure 9).

## 3. Discussion

In our study, we demonstrated that the FEE has antioxidant, antiaging, and anti-inflammatory activity. Simultaneously, some phenolic acids containing in FEE penetrate through the human skin and accumulate in it. 

### 3.1. Chemical Characterization of the FEE and Its Antioxidant Capacity 

The GC-MS analysis showed the contents of 11 main compounds. Fatty acids were the main components. The presence of methyl esters of fatty acids was also confirmed in the extracts of dried and fresh leaves of *E. angustifolium* [16] and essential oils from *E. angustifolium* [17] and *E. hirsutum* [18]. Canli et al. also identified fatty acids in ethanol extracts of *E. montanum* [19]. In our study, other compounds, as 2-methyl-Z,Z-3,13-octadecadienol and glyoxylic acid, were also identified in the samples. 2-methyl-Z,Z-3,13-octadecadienol was also the primary component of methanol extract from *Lagenaria breviflora fruit* [20], as well as of the aqueous stem bark extract of *Khaya senegalensis* [21]. At the same time, other similar organic compounds of the aldehydes group, among other 2-decanal, hexanal, octanal, N-nonanal, and alcohols, were identified in *E. angustifolium* by Jariene et al. [22]. 

In our study, phenolic acids such as ChA, GA, 4-HB, 3-HB, 3,4-DHB, and CA were identified by HPLC, and GA was found in a considerable amount. Some phenolic acids, like GA, ChA, 3,4-DHB, and CA, were also found in the herbs of *E. angustifolium* by Zagórska-Dziok et al., Nowak et al., Ruszová et al., and Lasinskas et al. [3,10,23,24], whereas Zagórska-Dziok et al. and Shikov et al. found the highest GA compared to the other phenolic acids [3,25]. On the other hand, a lower content of GA and ChA in *E. angustifolium* compared to other polyphenol compounds was identified by Jariene et al. [22]. GA was also identified in *E. hirsutum* [26,27]. The phenolic acids contained in *E. angustifolium*, belonging to the group of hydroxybenzoic acids (GA, 4-BH, 3-HB, and 3,4-DBH), as well as to the group of hydroxycinnamic acids (ChA and CA), have antibacterial, anti-atherosclerotic, anticarcinogenic, and anti-inflammatory properties [28,29,30,31,32]. They are known to be scavengers of various oxygen species, even as toxic as the HO^•^ radical and singlet oxygen [13]. This activity plays a vital role in skin regeneration [33] and prevents skin elasticity from losing tone, inhibiting the aging process. Therefore, the antioxidant activity of phenolic acids is beneficial in cosmetic and dermatologic formulations. The research studies confirm the positive correlation between antioxidant activity and phenolic acid content or other polyphenols [34,35,36]. Our research also demonstrated the antioxidant activity of the FEE, which was confirmed by other authors [16,23,37,38,39,40,41,42]. Similar results were also observed in other *Epilobium* varieties, such as *E. parviflorum*, *E. roseum, E. hirsutum, E. montanum, E. palustre*, and *E. adenocaulon* [19,27,42]. In our study, the antioxidant activity carried in the concentration range from 10 μg/mL to 1000 μg/mL for 30 min, whereas the measurements were performed at five-minute intervals. For most of the tested concentrations, an increase in antioxidant activity was observed slightly over time. Interestingly, the analyzed extract showed antioxidant activity from the very beginning of the experiment. Zagórska-Dziok et al. obtained a similar result when analyzing the water–ethanol extract of *E. angustifolium* [3]. Secondary metabolites, such as phenolic acids and flavonoids, are the dominant active compounds responsible for the antioxidant properties of the *Epilobium* genus [3]. These substances neutralize free radicals, which, in large amounts, may be involved in the pathogenesis of some skin disorders [4]. The multiactivity of polyphenols is also similar to others in their anti-inflammatory activity. For example, flavonoids inhibit the oxidative processes of membrane lipids, which lead to arachidonic acid release. Due to their affinity to proteins and metals, chelation flavonoids inactivate 5-lipoxygenase and cyclooxygenase, playing a key role in arachidonic acid transformation into proinflammatory leukotrienes (LTs) and prostaglandins [43]. Another essential substance in plant extracts is the assimilation pigments. Chlorophyll may also enhance the antioxidant properties [44]. In our study, we showed the total polyphenol and flavonoids and the assimilation pigments contents in FEE. The high total polyphenol and flavonoid contents in the *E. angustifolium* extracts were confirmed by Lasinskas et al., Shikov et al., and Zagórska-Dziok et al. [3,23,25]. 

### 3.2. Anti-Collagenase and Anti-Elastase Activity 

In the next stage of the research, the potential antiaging properties of FEE were assessed. For this purpose, an analysis of the possibility of inhibiting the activity of two enzymes, elastase, and collagenase, which are closely involved in the skin-aging processes, was carried out. It is mainly because these metalloproteinases break down the basic structural proteins of the skin, such as collagen and elastin [45]. During skin-aging processes, the increased activity of matrix metalloproteinase-1 (MMP-1), matrix metalloproteinase-8 (MMP-8), and matrix metalloproteinase-13 (MMP-13) from the collagenase family contributes to the breakdown of these skin proteins, leading to degenerative changes in the extracellular matrix, as well as causing the appearance of wrinkles, sagging skin, and accelerated skin aging [46]. Our study demonstrated that the FEE shows the possibility of inhibiting the activity of these enzymes in a concentration-dependent manner. The most promising effect was obtained using the extract at the concentration of 1000 µg/mL, in which the inhibition of elastase activity by 49.1% and collagenase by 59.8% was achieved. Comparing the obtained results to the level of inhibition of the activity of these enzymes by their commonly known inhibitors, which were also used in this work, it can be concluded that this extract can be seen as a source of compounds with potential antiaging properties, protecting the elastin and collagen fibers from degradation. The possibility of inhibiting skin aging processes by other types of *E. angustifolium* extracts was also noticed by other authors [3,28]. Karakaya et al. showed that the extract of ethyl acetate from the aerial part of *E. angustifolium* showed wound healing, among others, by inhibiting collagenase enzymes, while the aqueous extract of *E. angustifolium* showed strong elastase inhibitory activities [28]. The naturally organic compounds found in *E. angustifolium* extracts include both primary and secondary metabolites. The high contents of plant compounds with a broad spectrum of activity, confirmed by us in chromatographic analyses, certainly play a significant role in inhibiting the activity of the metalloproteinases mentioned above. Based on the literature data, this effect may result from the actions of such plant compounds as flavonoids, tannins, phenolic acids, terpenoids, or tocopherols [47,48]. The literature data also showed that some flavonoids, especially flavonols, can prevent collagen degradation by inhibiting collagenase activity in the skin. The authors indicated that flavonols are more potent inhibitors than flavones or isoflavones, which may be related to the C-3 hydroxyl substitution. Moreover, this activity is observed in intact skin and inflamed or photoaging skin due to UV radiation [49]. The presence of carbonyl and hydroxyl groups in the flavonoid molecules contained in numerous plant extracts allows them to form complexes with ions of various metals. As a result, they can also bind to metalloenzymes such as elastase and collagenase and significantly influence various metabolic pathways in our body [50]. To maintain the proper physiological state of the body, it is crucial to maintain a constant balance between the activity of MMPs and the level of endogenous compounds that can inhibit the activity of these enzymes [46], because the overexpression of both collagenase and elastase is directly related to the skin-aging process [45]. Other authors have demonstrated that plant flavonoids such as quercetin and kaempferol can reduce MMP-1 expression by inhibiting activator protein-1 (AP-1) activation. However, they indicated that the cellular mechanisms of metalloproteinase inhibition differ depending on the chemical structure of the flavonoids [51]. Moreover, the literature data indicate that elastase can stimulate other matrix metalloproteinases that accelerate the proteolytic degradation of biomolecules that are part of the extracellular matrix, such as fibronectin, proteoglycans, type IV collagen, vitronectin, laminin, chondroitin sulfate, and the basic myelin protein, which additionally adversely affects the skin condition [52,53]. Various plant compounds with proven antiaging properties, such as gallic acid, chlorogenic acid, and caffeic acid in the tested extract, and various volatile compounds are likely to play an important role in their antiaging properties [48,50,54,55]. It should be noted, however, that the activity of the extract cannot be attributed to the activity of a single compound or even a single class of compounds, because the stimulating or inhibiting activity of the tested metalloproteinases may be the result of synergism or antagonism between individual plant compounds [46]. Thus, the antiaging effect of FEE is probably the result of the interaction of several compounds present in this extract.

### 3.3. Anti-Inflammatory Potential

Inflammation is associated with many skin diseases, hence a recent tendency to look for natural plant compounds that can replace synthetic drugs, including nonsteroidal anti-inflammatory drugs, in combating both the causes and effects of inflammatory conditions [56]. Although plant substances sometimes require a more extended period of use, they are characterized by much greater safety and fewer side effects. Many authors have indicated that many of them show better outcomes than commonly used medicinal substances, and their uses may become an effective treatment strategy for chronic inflammation [56,57]. Therefore, as part of this work, the anti-inflammatory properties of the prepared extract were investigated by assessing the possibility of inhibiting lipoxygenase activity and the effect on protein denaturation. Lipoxygenase is an oxidative enzyme with an active non-heme iron atom in its active site and is involved in regulating the inflammatory response. It is associated with proinflammatory mediators leukotrienes or anti-inflammatory mediators known as lipoxins [58]. These enzymes catalyze the oxidation of polyunsaturated fatty acids such as linoleic and arachidonic acids. The effect of lipid oxidation is the initiation of the subsequent biological reactions and the activation of various cell signaling mechanisms [59]. In our study, the performed analyses showed that the effect of the extract on lipoxygenase activity was dose-dependent. The most potent inhibition was obtained for the extract at a concentration of 1000 µg/mL, where it reached 68.2%, but at a concentration of only 100 µg/mL, the inhibition reached over 40%. In another study assessing the possibility of inhibiting bovine serum albumin denaturation by the *E. angustifolium* extract, the potential anti-inflammatory properties of the studied extract were also indicated. The conducted analyses showed that the 1000-µg/mL concentration of FEE could inhibit BSA denaturation by 67.7%, which may potentially play an important role in preventing or reducing inflammation. Protein denaturation significantly affects its spatial structure and the loss of its biological properties. According to the authors, this denaturation may contribute to the formation of various inflammatory diseases. Therefore, the ability of plant substances to prevent protein denaturation may also help prevent inflammation [60,61]. Although the conducted analyses showed that the extract obtained by us has slightly lower anti-inflammatory properties than the commonly used acetylsalicylic acid or the well-known compound with anti-inflammatory properties—quercetin—the obtained results may be an impulse to perceive the studied extract as a valuable raw material with anti-inflammatory properties. Considering the numerous side effects associated with the use of drugs such as acetylsalicylic acid, this extract may prove to be a much safer alternative [62]. Moreover, the literature data showed that there is a great chance for synergism between the active ingredients contained in them and obtaining a much more favorable effect in the case of using whole-plant extracts, because when individual components are isolated, this effect may be lost or unnoticeable. Such synergism has been confirmed in various studies, including anti-inflammatory studies [57,63,64]. It should be noted that one component may also reduce the biological activity of the other. At the same time, the promising results obtained in this study indicated that the entire extract can be seen as a mixture with anti-inflammatory properties. The content of a broad spectrum of biologically active compounds in the obtained extract, confirmed in chromatographic analyses, certainly influences the anti-inflammatory effect. Many of them have been indicated by other authors as compounds with anti-inflammatory properties [65,66].

### 3.4. Penetration Skin 

The active substances contained in plants can play an important role in the regeneration of the epidermis and the underlying layers. The more important components showing such activity include phenolic acids. The assessment of their skin penetration is an important element in modeling cosmetic forms containing plant extracts [66,67]. The plant extracts generally have greater antioxidant activities than isolated pure compounds because of synergism between components of whole plant extract [67]. However, it is important to determine the degree of penetration through the skin of the active substances. The penetration degree of these substances may be different, depending on the compound’s physical and chemical properties, such as lipophilicity, structure, and polarity [68,69]. To achieve the benefit action of phenolic acids in the skin, they should be released from the topical formulation and permeate the SC, which is the main skin barrier. It is a thin membrane consisting primarily of cornified epidermal cells, while the main components are lipids, namely ceramides, cholesterol and its esters, and fatty acids [15]. Moreover, biological factors such as the age and condition of the skin have a great influence. To obtain the good benefits of “natural” preparation, the compounds they contain must reach deeper layers of the skin to perform their biological activities [1]. To increase the active compounds’ permeability, various vehicles can promote or reduce skin penetration [70]. The correct selection of the semisolid base and its ingredients is essential for the effective penetration of the bioactive ingredients [71]. The vehicle used in the case of herbal preparations can significantly influence the release and penetration of the active substances through the skin. Our study estimated the in vitro penetration of FEE in two vehicles, such as a hydrogel and an emulsion, wherein we used the plant extract in two concentrations—2.5% and 5%. As suggested by Bertges et al., the concentration of plant extracts in dermatological or cosmetic preparations should be up to 5%. The incorporation of plant extracts at concentrations above 5% to the vehicle could lead to physical instability of the formulation [67]. Our study showed that phenolic acids penetrated with hydrogel much better in comparison to the emulsion. The highest skin penetration was observed with H-5FEE. Similarly, Žilius et al. showed the highest penetration of phenolic acids from a hydrogel containing a propolis extract. These authors showed that, after 8 h, hydrophilic gel released nearly the whole amount of phenolic acids such as coumaric, caffeic, and ferulic, while only up to 5% and 22% of phenolic compounds were released from ointment and/or cream, respectively. These authors suggested that preparation with lower oil causes promoted higher release rates of phenolic compounds. The higher viscosity of the emulsions, containing oil content, hampers phenolics diffusion and, finally, leads to the lower release and permeation rates of the phenolic compounds [72]. In our study, from among the studied phenolic acids, GA and 4-HB penetrated to a higher degree than the others. For H-5FEE, the cumulative mass of phenolic acids in the acceptor fluid was significantly higher than the other vehicles. Interestingly, the cumulative mass of GA, ChA, and 4-HB did not differ significantly between H-2.5FEE and E-5FEE. No CA was detected in the acceptor fluid, except for the use of a 5% hydrogel. Similarly, the very low penetration of CA through the pigskin was confirmed by Bertges et al. These authors analyzed the release of phenolic acids from a hydrogel containing 5% coffee seed extract [67]. On the other hand, Marti-Mesteres et al. showed high penetrations of CA through pigskin. However, these authors applied CA as a pure compound dissolved in propylene glycol at the time penetration of 72 h [73], while, in our previous studies, CA penetrated through human skin from a 70% (*v*/*v*) ethanol solution [10]. Ethanol is a promoter of transepidermal transport, which affects the effectiveness of active substance penetration into the skin [74,75]. The in vitro monitoring of active substances release is a useful tool for producing cosmetic or pharmaceutical topical products such as emulsions or hydrogels [15,76]. Compounds that act on deeper skin layers have to penetrate the stratum corneum to reach the cells localized in the lower strata of the epidermis, the dermis, or even the cutaneous microcirculation. On the other hand, cosmetic formulations are generally considered safer if the permeation of their main compounds is limited to the upper layers of the skin and does not reach the microcirculation [1]. In our study, all the phenolic acids accumulated in the human skin, wherein significant differences depending on the vehicle used were observed. After applying E-5FEE, the skin accumulated significantly more ChA, 4-HB, and 3,4-DHB compared to the other vehicles. For example, in some analgesic drugs, a faster penetration is preferable to achieve a rapid therapeutic effect. Whereas, in the case of plant substances such as phenolic acids, their greater accumulation in the skin is preferred, where they will show the effect of, among others, antiaging [76].

## 4. Materials and Methods 

### 4.1. Chemicals

2,2-diphenyl-1-picrylhydrazyl (DPPH), 6-hydroxy-2,5,7,8-tetramethylchroman-2carboxylic acid (Trolox), 2,2′-azino-bis(3-ethylbenzothiazoline-6-sulfonic acid) (ABTS), 2,4,6-tripyridyl-s-triazine (TPTZ), 3,4-dihydroxybenzoic acid, chlorogenic acid, caffeic acid, acetylsalicylic acid, bovine albumin serum (BSA), linoleic acid sodium salt (≥98%), quercetin, rutin, and soybean lipoxygenase were purchased from Sigma Aldrich (Steinheim am Albuch, Germany); Folin–Ciocalteu reagent, gallic acid, 4-hydroxybenzoic acid, 3-hydroxybenzoic acid, disodium phosphate, propylene glycol, and potassium dihydrogen phosphate from Merck, Darmstadt (Germany); sodium acetate anhydrous, potassium persulfate, potassium acetate, 99.5% acetic acid, 36% hydrochloric acid, sodium chloride, potassium chloride, ethanol, methanol, acetone, and phosphate-buffered saline (PBS; pH 7.00 ± 0.05) were from Chempur (Piekary Śląskie, Poland), whereas acetonitrile for HPLC was from J.T. Baker (Berlin, Germany). Hydroxyethylcellulose, 1,10-phenanthroline, and succinyl-alanyl-alanyl-prolyl-valine chloromethylketone (SPCK, Abcam, Cambridge, MA, USA) were used as received. All reagents were of analytical grade. 

### 4.2. Plant Materials

The plant materials were collected during the flowering phase in July in Poland in 2020 (N 53°23′18″, E 14°28′56″) from the natural state. The plants were selected randomly from different, near-located places. Six samples were harvested and combined into one collective sample. The aerial part of *E. angustifolium* herb was harvested during the massive blooming period [16,77]. The plant material was dried at room temperature in a well-ventilated area to a constant weight [16]. Samples were deposited in the plant material storage room (No. EAE-AM2020-01) at the Chair and Department of Cosmetic and Pharmaceutical Chemistry of the Pomeranian Medical University in Szczecin, Poland. The plant material was ground in the grinder and sieved using a circular-hole screen (8-mm mesh). Five grams of dried raw material were extracted with 100 mL 70% (*v*/*v*) ethanol for 60 min in an ultrasonic bath at a frequency of 40 kHz. Then, obtained extracts were collected and filtered through Whatman filter paper No. 10, codified EEA03 (Merck, Darmstadt, Germany). After filtration, the FEE was evaporated under reduced pressure at 40 °C. A stock solution at the concentration of 100 mg/mL was prepared from the dried extracts in 70% (*v*/*v*) ethanol. The samples were stored in the dark at 4 °C until further analysis. 

### 4.3. Determination of Biologically Active Compounds 

#### 4.3.1. Determination of Bioactive Compounds by GC-MS and HPLC

Qualitative chemical analyses were performed using a GC–MS system comprised of TRACE GC series apparatus with a VOYAGER mass detector (Agilent, Santa Clara, CA, USA) using a DB5 capillary column (30 m × 0.25 mm × 0.5-µm film thickness). The carrier gas was helium at a constant flow of 1.0 mL/min, sample chamber temperature of 240 °C, and a detector voltage of 350 V. The sample partition coefficient in the dispenser was 20, the volume of dispensed sample was 0.3 μL, and the ion mass range was 25–350 mV/z. The oven was held at 50 °C (2 min), then increased by 10 °C/min to 310 °C, and then cooled to 50 °C. 

The concentration of test compounds in the FEE was determined by high-performance liquid chromatography (HPLC), using the HPLC system from Knauer (Berlin, Germany). The tested components were separated on a 125 mm × 4 mm column containing Hyperisil ODS, particle size 5 µm. The mobile phase consisted of acetonitrile, 1% acetic acid, and MeOH (45:45:10 by vol.), and the flow rate was 1 mL/min. Twenty cubic millimeters of the sample were injected onto the column. The spectrophotometric detector was set at 280 nm. The correlation coefficient of the calibration curve was 0.9964 (y = 277926x + 0.226, Rt-2.868 min) for gallic acid, 0.9992 for chlorogenic acid (y = 53905x + 9.831, Rt −15.589 min), 0.999 for 4-hydroxybenzoic acid (y = 26889x + 3.5605, Rt −8.786 min), 0.999 for 3-hydroxybenzoic acid (y = 15214x + 0.5775, Rt −8.003 min), 0.999 for 3,4-dihydroxybenzoic acid (y = 78007x − 1.1925, Rt −5.191 min), and 0.9994 for caffeic acid (y = 67950x + 5.141, Rt −11.260). The extracts were diluted 10-fold before injection. All samples were analyzed three times. 

#### 4.3.2. Total Phenolic Content Determination (TPC)

The total polyphenol content was determined with the Folin–Ciocalteu method, as described previously [38]. Shortly, to 0.15 mL of the studied sample, 0.15 mL of tenfold diluted Folin–Ciocalteu reagent, 1.35 mL of 0.01-M sodium carbonate solution, and 1.35 mL of water were added and mixed. The cuvette was sealed with a stopper and then incubated for 15 min at room temperature. After this time, the spectrophotometric measurement was carried out at 765 nm. Gallic acid (GA) was applied as a standard, and the results were expressed as gallic acid equivalents (GA) in mg GA/g dry mass (DWE). Three independent measurements were made.

#### 4.3.3. Total Flavonoids Content Determination (TFC)

The assessment of the total flavonoid content in the analyzed extracts was performed spectrophotometrically by the method described by Zagórska-Dziok et al., with some modifications [3]. Shortly, the reaction mixture was prepared to contain 80% C_2_H_5_OH, 10% Al(NO_3_)_3_ × 9 H_2_O, and 1 M C_2_H_3_KO_2_. Then, 2400 µL of the previously prepared reaction mixture was mixed with 600 µL of the tested extract sample. After incubation for 40 min at room temperature, the absorbance was measured at λ = 415 nm with a spectrophotometer. Rutin (RR) was applied as a standard, and the results were expressed as rutin equivalents (RR) in mg RR/g dry mass (DWE). Three independent measurements were made.

#### 4.3.4. Determination of Chlorophyll Content

The chlorophyll content of FEE was determined by spectrophotometry by the method described by Liang et al. and Zagórska-Dziok et al. [48,78]. Stock solution of dry extracts at a concentration of 100 µL/mL in 80% acetone was prepared. The absorbance of the solutions was measured at λ = 645 nm and λ = 663 nm. The results were expressed as the content of chlorophyll a and b and the total content of chlorophyll (a + b), calculated in a mg/g dry mass extract (DWE). The chlorophyll content was calculated using the following equation:

Chlorophyll a = 12.7 (A_663_) − 2.69 (A_645_), Chlorophyll b = 22.9 (A_645_) − 4.68 (A_663_), Total chlorophyll (a + b) = 20.2 (A_645_) + 8.02 (A_663_), (1)

where:

A_663_—absorbance measured at wavelength 663;
A_645_—absorbance measured at wavelength 645.

Measurements were carried out in triplicate for each sample of extract.

### 4.4. Evaluation of the Antioxidant Activity

#### 4.4.1. DPPH Radical Scavenging Assay

The scavenging activity of DPPH stable free radicals was measured as described by Zagórska-Dziok et al. and Nowak et al., with modifications [3,38]. Shortly, an aliquot of 0.15 mL of the studied samples at the concentrations range of 10–1000 µg/mL was added and mixed with 2.85 mL of 0.3-mM DPPH radical solution dissolved in 96% (*v*/*v*) ethanol. The absorbance at 517 nm of the DPPH working solution was adjusted to 1.00 ± 0.02 with 96% (*v*/*v*) ethanol. Kinetics of the absorbance changes was done. The tests were carried for a period of 30 min. Measurements were performed at five-minute intervals. The first measurement after 5 min of incubation at room temperature was done. As a result, seven measurement points were realized for each concentration of the tested extract. The scavenging activity on the DPPH radical was expressed as an inhibition percentage using the following equation:

(2) $$\%DPPH\cdot scavenging=1-\frac{A_{s}}{A_{c}}\times 100\%$$

where:

*A_s_*—absorbance of the tested sample;
*A_c_*—absorbance of the control sample.

Measurements were carried out in triplicate for each sample of extract.

#### 4.4.2. ABTS Radical Scavenging Assay

The procedure applied to evaluate the ABTS radical scavenging activity was described previously by Nowak et al., with modifications [38]. Shortly, a 7-mM solution of ABTS (2,2′-azino-bis(3-ethylbenzothiazoline-6sulfonic acid)) in a 2.45-mM aqueous solution of potassium persulfate was used as a stock solution. After dissolving the components, the solution was incubated for 24 h in the dark at room temperature, then diluted with 50% (*v*/*v*) methanol to obtain a working solution of absorbance of 1.00 ± 0.02 at 734 nm. The antioxidant activity was measured as follows: 2.5 mL of working ABTS solution and 0.25 mL of a studied sample at the concentrations range of 10–1000 µg/mL were introduced into the spectrophotometric cuvette. As with the DPPH method, kinetics of the absorbance changes was done (see Section 4.4.1). The ABTS scavenging was calculated from the equation:

(3) $$\%\;ABTS\cdot scavenging=1-\frac{A_{s}}{A_{c}}\times 100\%$$

where:

*A_s_*—absorbance of the tested sample;
*A_c_*—absorbance of the control sample.

Measurements were carried out in triplicate for each extract sample.

### 4.5. Assessment of Antiaging Properties

#### 4.5.1. Determination of Anti-Elastase Activity

To assess the inhibition potential of matrix metalloproteinase, a fluorometric kit (Abcam, ab118971, Cambridge, MA, USA) was used to determine the activity of neutrophil elastase (NE). The analysis was based on the instructions provided by the manufacturer and the procedure previously described by Zagórska-Dziok et al. [3]. Fluorometric analyses were performed in a 96-well plate with black walls and clear bottom. The ability to inhibit the activity of elastase was determined for the FEE at the concentrations of 100, 500 and 1000 µg/mL. Initially, NE enzyme solutions, NE substrate, and inhibitor control (succinyl-alanyl-alanyl-prolyl-valine chloromethylketone; SPCK) were prepared according to the manufacturer’s guidelines. The NE solution was then added to all wells. The test samples, the inhibitor control, and the enzyme control (assay buffer) were added to the subsequent wells, and the samples were mixed thoroughly. All samples were analyzed in duplicate. The prepared plate was incubated in the dark at 37 °C for 5 min. Meanwhile, a reaction mixture was prepared by mixing assay buffer and NE substrate and added to each well. The fluorescence was then immediately measured at excitation wavelength λ = 400 nm and emission λ = 505 nm using a microplate reader (FilterMax F5, Thermo Fisher Scientific, Waltham, MA, USA). During the measurements, the kinetic mode was used (30 min at 37 °C). The influence of the tested samples on the NE activities was calculated from the equation:

(4) $$\%\;relative\;activity=\frac{\Delta \;RFU\;test\;inhibitor}{\Delta \;RFU\;enzyme\;control}\times 100\%$$

#### 4.5.2. Determination of Anti-Collagenase Activity

As part of this work, the ability to inhibit the activity of another matrix metalloproteinase—collagenase—was also assessed. For this purpose, a fluorometer kit (Abcam, ab211108, Cambridge, MA, USA) was used. The activity of this enzyme was assessed based on the manufacturer’s instructions and the procedure previously described by Nizioł-Łukaszewska et al. [10]. Analyses were carried out in a black 96-well plate with a clear flat bottom. The analyses were performed for the extracts at concentrations of 100, 500, and 1000 µg/mL. In the first step, collagenase (COL) was dissolved in a collagenase analysis buffer (CAB). Then, the enzyme solution prepared in this way was added to the test samples. Inhibitor controls were prepared by mixing a collagenase inhibitor (1,10-phenanthroline (80 mM)) with a collagenase and CAB buffer. An enzyme control was prepared by mixing diluted COL with CAB. A CAB buffer was used as the background control. The prepared samples were then incubated for 15 min at room temperature in the dark. Meanwhile, a reaction mixture was prepared by mixing collagenase substrate with CAB. This mixture was added to all the analyzed samples and mixed thoroughly. In the next step, fluorescence was measured at the excitation wavelength λ=490 nm and emission at λ=520 nm using a microplate reader (FilterMax F5, Thermo Fisher Scientific, Waltham, MA, USA). The measurements were performed in kinetic mode for 60 min at 37 °C. All samples were prepared in duplicate according to the manufacturer’s instructions. The ability to inhibit COL activity by FEE was calculated from the equation:

(5) $$\%\;relative\;COL\;inhibition=\frac{\mathrm{enzyme}\;\mathrm{control}-\mathrm{sample}}{\mathrm{enzyme}\;\mathrm{control}}\times 100\%$$

### 4.6. Determination of Anti-Inflammatory Properties

#### 4.6.1. Inhibition of Lipoxygenase Activity

The anti-inflammatory properties of FEE were assessed by determining the ability to inhibit lipoxygenase activity. For this purpose, the method described by Sarveswaran et al. [79] was used. Initially, the analyzed extract at concentrations of 100, 500, and 1000 µg/mL was mixed in a standard 96-well plate with 100-mM PBS and a soybean lipoxygenase solution (167 U/mL). The prepared plates were incubated at 25 °C for 10 min; after which, 10 µL of sodium linoleic acid salt was added to initiate the reaction. In the next step, the absorbance was measured every minute for 3 min at λ = 234 nm using a FilterMax F5 microplate reader (Thermo Fisher Scientific, Waltham, MA, USA). As a control, quercetin at analogous concentrations of 100, 500, and 1000 µg/mL was used. The samples were tested in 3 independent experiments, in which all samples were run in duplicate. The percentage of inhibition of lipoxygenase activity was calculated from the equation:

(6) $$\mathrm{inhibition}\;\mathrm{of}\;\mathrm{lipoxygenase}\;\mathrm{activity}=\frac{A_{c}-A_{s}}{A_{c}}\times 100\%$$

where:

A_s_—absorbance of the tested sample;
A_c_—absorbance of the control sample.

Measurements were carried out in triplicate for each sample of extract.

#### 4.6.2. Inhibition of Protein Denaturation 

In order to assess the anti-inflammatory properties of FEE, its ability to inhibit denaturation of BSA was assessed. For this purpose, the method described by Sarveswaran et al. [79] was used. Initially, 1000 µL of the test extract (at concentrations of 100, 500, and 1000 µg/mL) were mixed with 450 µL of a 5% aqueous BSA solution and 1400 µL of phosphate-buffered saline (PBS). The mixture was then incubated at 37 °C for 15 min. In the next step, it was heated to 70 °C for 5 min; then, the reaction solutions were cooled in an ice bath to 25 °C, and the absorbance was measured at λ = 660 nm using a DR600 UV-Vis spectrophotometer (Hach Lange, Wrocław, Poland). As a positive control, acetylsalicylic acid was used at analogous concentrations of 100, 500, and 1000 µg/mL. All samples were analyzed in triplicate from three independent experiments. The percentage of protein denaturation inhibition was calculated based on the equation:

(7) $$inhibition\;of\;denaturation=\frac{1-A_{s}}{A_{c}}\times 100\%$$

where:

A_s_—absorbance of the tested sample;
A_c_—absorbance of the negative control.

Measurements were carried out in triplicate for each sample of extract.

### 4.7. Hydrogel End Emulsion Preparation

**Figure 10:**
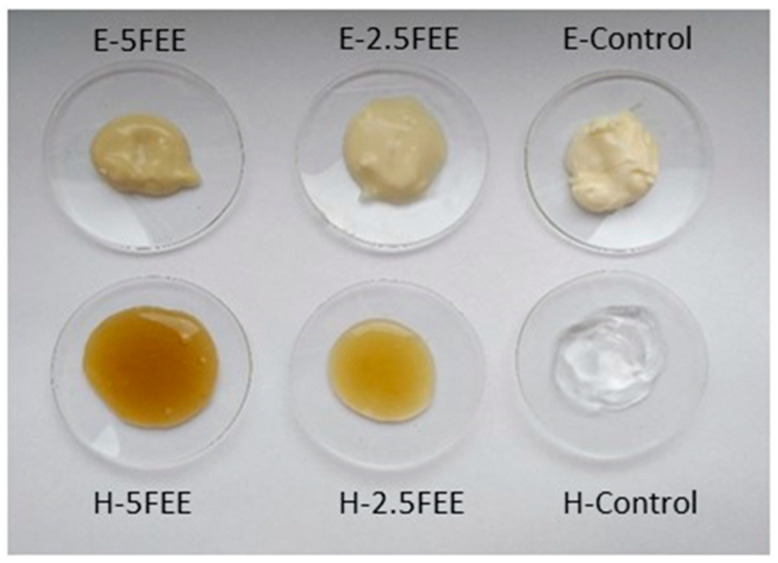
The hydrogel end emulsions with the dry extracts from *E. angustifolium* FEE.

The hydrogel was prepared according to a modified procedure by Zagórska-Dziok et al. [44]. Sequentially, an aqueous solution of hydroxyethyl cellulose (HEC) was prepared. HEC was added to water and mixed on a mechanical stirrer (ChemLand, Stargard, Poland) using a stirrer and stirring speed of 250 rpm. Then, the polymer solution was heated to 60 °C and then cooled to room temperature while constantly stirring. The emulsion (W/O) was prepared according to a modified method by Suñer-Carbó et al. [80]. Sequentially, the emulsion was prepared by a slow addition of the oil phase to the aqueous phase at a temperature of 80 °C under continuous stirring at 250 rpm. The oil phase was lipophilic components such as Biobase, grape seed oil, and bee wax. Biobase is a commercial preparation containing glyceryl stearate, cetearyl alcohol, and sodium stearoyl lactylate. The water phase consisted of propylene glycol, HEC, and water. The resulting mixture was stirred until a homogeneous emulsion was completely formed. Dry extracts of *E. angustifolium* were added to the hydrogel and emulsion by dissolving them in a propane glycol solution [78]. The dissolved dry plant extracts were added after both vehicles cooled down. Two hydrogels were obtained, containing 2.5% (H-2.5FEE) and 5% FEE (H-5FEE). Similarly, two emulsions were obtained: E-2.5FEE and E-5FEE, respectively (Figure 10). The compositions of the hydrogels and emulsion are shown in Table 5.

### 4.8. In Vitro Skin Permeation Studies

The permeation experiments were performed in the Franz diffusion cells (SES GmbH Analyse Systeme, Bechenheim, Germany) with a diffusion area of 1 cm^2^. The acceptor chamber was filled with PBS solution (pH 7.4). In each diffusion unit, a constant temperature of 32.0 ± 0.5 °C [76] was maintained via a thermostat (VEB MLW Prüfgeräte-Werk type 3280, Leipzig, Germany). The acceptor chamber content was stirred with a magnetic stirring bar at the same speed for all cells. The donor chamber volume was 2 mL, and the volume of the acceptor chamber was 8 mL. Human abdominal skin obtained after plastic breast surgery was used. The study was approved by the Ethical Committee of Pomeranian Medical University in Szczecin (KB0012/02/18). The skin of 0.5 mm in thickness was divided into about 2 cm × 2 cm pieces. Next, the skin samples were wrapped in aluminum foil and stored in a freezer at −20 °C until use, not longer than three months. This frozen storage time was safe to keep skin barrier properties [81]. On the day of the experiment, the skin samples were slowly thawed at room temperature and were hydrated by PBS, pH 7.4 [82,83,84,85]. Undamaged pieces of skin were placed in a Franz diffusion cell between the donor and acceptor chambers. The integrity of the skin was measured. For this purpose, an LCR meter 4080 (Voltcraft LCR 4080, Conrad Electronic, Kraków, Poland), operated in parallel mode at an alternating frequency of 120 Hz (error at kΩ values <0.5%), was used. The tips of the measuring probes were immersed in the donor and acceptor chambers and filled with PBS (pH 7.4) as described previously [85,86]. Only skin samples with impedance >3 kΩ were used. These values are similar to the electrical resistance of human skin [87]. Thereafter, a defined dose (1 g) of the vehicle was applied to the skin’s outer side. All donor chambers were closed with plastic stoppers. The penetration study was carried out for 24 h. At the time points of 1, 2, 3, 5, 8, and 24 h, 0.5 mL of acceptor samples were withdrawn, and the chamber was refilled with the same volume of a fresh buffer of the same pH. The phenolic acid concentrations in the acceptor phase were measured by the HPLC method. The cumulative mass (µg) of each phenolic acid studied was calculated based on the obtained concentration. After the end experiment, diffusion cells were disassembled, and the skin samples were analyzed for the contents of the selected phenolic acids. The accumulation of the tested compounds in the skin after penetration was determined using a modification of the method described by Haq and Michniak-Kohl [82]. After 24 h of the experiment, each skin sample was removed. In order to remove the vehicle residues, they were carefully rinsed with 0.5% sodium lauryl sulfate solution. The skin was then cut around the diffusion area (1 cm^2^) and dried at room temperature. Each of the 1-cm^2^ skin samples was cut into small pieces, placed in 2-mL methanol, and incubated for 24 h at 4 °C. After this time, skin samples were homogenized for 3 min using a homogenizer (IKA^®^T18 digital ULTRA TURRAX, Staufen, Germany). The homogenate was centrifuged at 3500 rpm for 5 min. The supernatant was collected for the subsequent HPLC and spectrophotometric analyses, with pure methanol applied as a control. Accumulation of the phenolic acids in the skin was calculated by dividing the amount of the substances remaining in the skin by the mass of the skin sample and was expressed as the mass of phenolic acid per mass of the skin (µg/g). Based on the phenolic acid permeation profiles, the permeation parameters such as the steady-state permeation flux (JSS), the diffusion coefficient (KP), and the time required to reach steady-state permeation (lag time—LT) were determined and compared depending on the type of vehicle and the concentration of FEE in this vehicle. The steady-state fluxes (JSS) of phenolic acid through the skin were calculated from the slope of the plot of cumulative mass in the acceptor phase over time and were expressed as the amount of compound per skin area and time (μg cm^−2^ h^−1^). The lag time was determined by extrapolating the equation, and KP was a quantitative measure of the rate at which a molecule can cross the skin. 

### 4.9. Statistical Analysis

The results were presented as the mean ± standard deviation (SD). A one-way analysis of variance (ANOVA) was used. The significance of differences between individual groups was evaluated with Tukey’s test (α < 0.05). Statistical calculations were done using Statistica 13 PL software (StatSoft, Kraków, Poland). 

## 5. Conclusions

The analyses of the *E. angustifolium* extract carried out as part of this work indicated that the analyzed plant was a valuable source of natural compounds with a large spectrum of activity. The FEE contained many active substances, including phenolic acids, Moreover, the antioxidant activity, measured by the DPPH and ABTS methods, of the studied extracts was observed, as well as the anti-inflammatory potential of the FEE. Due to the fact that compounds of natural origin are currently sought to replace synthetic substances in preparation for skin care and various skin disease treatments, two vehicles, i.e., emulsion and hydrogel, containing the tested extract were prepared. In the performed test, the penetration of the selected phenolic acids and their accumulation in the skin were observed. It was found that the phenolic acids contained in the hydrogel penetrated to a higher degree compared to emulsion. The obtained results could indicate the possibility of using *E. angustifolium* as an ingredient, for example, in cosmetics and pharmaceutics applied to the skin, due to its antiaging and anti-inflammatory activity.

## Figures and Tables

**Table 1:** Concentrations of phenolic acids of the ethanolic extracts from *E. angustifolium* (FEE). Values are the mean of three replicate determinations (*n* = 3).

Chemical Compounds	(mg/100 mL)
chlorogenic acid (ChA)	22.03 ± 0.19
gallic acid (GA)	113.07 ± 5.03
4-hydroxybenzoic acid (4-HA)	29.72 ± 0.25
3-hydroxybenzoic acid (3-HB)	1.66 ± 0.50
3,4-dihydroxybenzoic acid (3,4-DHB)	5.91 ± 0.36
caffeic acid (CA)	0.92 ± 0.08

**Table 2:** Total contents of the polyphenols (TPC) and flavonoids (TFC) assimilation pigments in the ethanolic extracts from *E. angustifolium* (FEE). The contents of the phenolic compounds were determined using gallic acid (GA) and flavonoids using quercetin (QE). Values are the mean of three replicate determinations (*n* = 3).

TPC (mg GA/g DWE)	TFC (mg RR/g DWE)	Assimilation Pigments (mg/g DWE)		
		Chlorophyll a	Chlorophyll b	Total Chlorophyll
22.15 ± 0.13	3.37 ± 0.01	0.58 ± 0.02	0.15 ± 0.00	0.74 ± 0.02

DWE—dry weight of extract, GA—Gallic acid, and RR—Rutin.

**Table 3:** Phenolic acids concentration in the acceptor fluid after 24-h penetration through the human skin after an application of dry ethanolic extracts from *E. angustifolium* (FEE) in two vehicles. Values are the mean of three replicate determinations (*n* = 3).

Phenolic Acid	Cumulated Mass (µg)			
	Emulsion		Hydrogel	
	E-2.5FEE	E-5FEE	H-2.5FEE	H-5FEE
ChA	3.03 ± 0.35 ^c^	5.98 ± 0.9 ^b^	5.82 ± 0.53 ^b^	10.27 ± 1.08 ^a^
GA	5.59 ± 0.44 ^c^	10.88 ± 0.65 ^b^	12.69 ± 1.13 ^b^	18.44 ± 1.13 ^a^
3-HB	nd	nd	nd	nd
4-HB	3.31 ± 0.36 ^c^	8.17 ± 0.54 ^b^	6.21 ± 1.38 ^c^	16.12 ± 0.80 ^a^
3,4-DHB	2.13 ± 0.03 ^d^	7.19 ± 0.57 ^b^	5.51 ± 0.42 ^c^	10.78 ± 0.93 ^a^
CA	nd	nd	nd	0.89 ± 0.05
Sum of phenolic acids	14.06	32.24	30.23	56.52

Different letters indicate significant differences between all used vehicles (α = 0.05), nd—no detected.

**Table 4:** The parameters characterizing phenolic acid transport through the human skin after the application of ethanolic extracts from *E. angustifolium* (FEE) in two vehicles.

	**Emulsion**					
**Phenolic Acid**	**J_SS_** **(μg·cm^−2^·h^−1^)**		**Lag Time** **(h)**		**K_P_·10^−8^** **(cm·h^−1^)**	
	**E-2.5FEE**	**E-5FEE**	**E-2.5FEE**	**E-5FEE**	**E-2.5FEE**	**E-5FEE**
ChA	0.22	0.48	0.178	<0.001	4.00	4.45
GA	0.48	0.93	0.844	0.870	0.55	5.32
3-HB	nd	nd	nd	nd	nd	nd
4-HB	0.42	0.94	1.941	1.767	9.65	10.82
3,4-DHB	0.08	0.40	<0.001	<0.001	1.51	7.95
CA	nd	nd	nd	nd	nd	nd
	**Hydrogel**					
**Phenolic Acid**	**J_SS_** **(μg·cm^−2^·h^−1^)**		**Lag Time** **(h)**		**K_P_·10^−8^** **(cm·h^−1^)**	
	**H-2.5FEE**	**H-5FEE**	**H-2.5FEE**	**H-5FEE**	**H-2.5FEE**	**H-5FEE**
ChA	0.78	1.09	1.049	<0.001	14.07	9.86
GA	0.77	1.19	0.500	<0.001	0.88	0.68
3-HB	nd	nd	nd	nd	nd	nd
4-HB	0.56	1.63	1.524	1.689	12.88	18.80
3,4-DHB	0.34	0.65	2.000	<0.001	6.64	12.75
CA	nd	0.29	nd	5.001	nd	5.64

nd—no detected.

**Table 5:** Compositions of the prepared hydrogels and emulsions used in the penetration study.

Ingredient	Emulsion		Hydrogel	
	E-2.5FEE	E-5FEE	H-2.5FEE	H-5FEE
FEE *	2.5	5.0	2.5	5.0
HEC *	-	-	2	2
Glycol propanol *	20	20	20	20
Biobase *	6	6	-	-
Grape seed oil	20	20	-	-
Bee wax *	7	7	-	-
Water up to *	100	100	100	100

* The amount of components is expressed in g; FEE—dry extract of *E. angustifolium.* HEC—hydroxyethyl cellulose.

## Data Availability

The data presented in this study are available in this article.

## Sample Availability

Samples of the compounds are available from the authors.
